# Photo-responsive polymeric micelles and prodrugs: synthesis and characterization

**DOI:** 10.1039/c8ra04580a

**Published:** 2018-08-17

**Authors:** Shiu-Wei Wang, Yin-Ku Lin, Jia-You Fang, Ren-Shen Lee

**Affiliations:** Division of Natural Science, Center of General Education, Chang Gung University 259 Wen-Hwa 1st Road, Guishan Dist. Tao-Yuan 33302 Taiwan shen21@mail.cgu.edu.tw; Department of Traditional Chinese Medicine, Chang Gung Memorial Hospital at Keelung Keelung Taiwan; Graduate Institute of Natural Products, Chang Gung University Tao-Yuan Taiwan

## Abstract

Bio-recognizable and photocleavable amphiphilic glycopolymers and prodrugs containing photodegradable linkers (*i.e.* 5-hydroxy-2-nitrobenzyl alcohol) as junction points between bio-recognizable hydrophilic glucose (or maltose) and hydrophobic poly(α-azo-ε-caprolactone)-grafted alkyne or drug chains were synthesized by combining ring-opening polymerization, nucleophilic substitution, and “click” post-functionalization with alkynyl-pyrene and 2-nitrobenzyl-functionalized indomethacin (IMC). The block-grafted glycocopolymers could self-assemble into spherical photoresponsive micelles with hydrodynamic sizes of <200 nm. Fluorescence emission measurements indicated the release of Nile red, a hydrophobic dye, encapsulated by the Glyco-ONB-P(αN_3_CL-*g*-alkyne)_*n*_ micelles, in response to irradiation caused by micelle disruption. Light-triggered bursts were observed for IMC-loaded or -conjugated micelles during the first 5 h. Following light irradiation, the drug release rate of IMC-conjugated micelles was faster than that of IMC-loaded micelles. Selective lectin binding experiments confirmed that glycosylated Glyco-ONB-P(αN_3_CL-*g*-alkyne)_*n*_ could be used in bio-recognition applications. The nano-prodrug with and without UV irradiation was associated with negligible levels of toxicity at concentrations of less than 30 μg mL^−1^. The confocal microscopy and flow cytometry results indicated that the uptake of doxorubicin (DOX)-loaded micelles with UV irradiation by HeLa cells was faster than without UV irradiation. The DOX-loaded Gluco-ONB-P(αN_3_CL-*g*-PONBIMC)_10_ micelles effectively inhibited HeLa cells' proliferation with a half-maximal inhibitory concentration of 8.8 μg mL^−1^.

## Abstract 2

Bio-recognizable and photocleavable amphiphilic glycopolymers and prodrugs containing photodegradable linkers as junction points between hydrophilic glycose and hydrophobic poly(α-azo-ε-caprolactone)-grafted alkyne or drug chains were synthesized.

## Graphical abstract

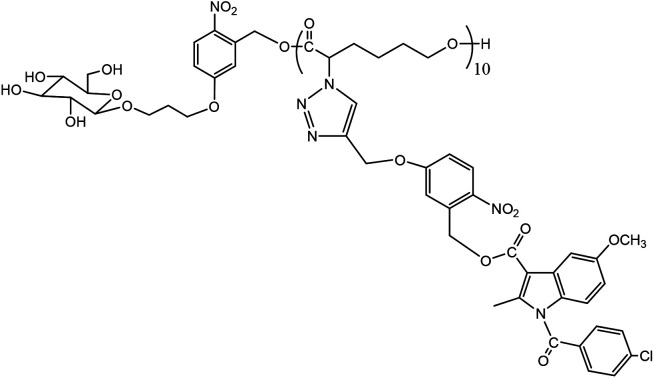

## Introduction

In recent years, multifarious drug delivery systems (*e.g.*, polymeric micelles, polymer drug conjugates, and polymeric nanoparticles) have been developed to address the problems associated with drug molecules, such as low aqueous solubility, short plasma circulation time, rapid *in vivo* degradation and systemic toxicity.^1^ Compared with free drugs, drugs incorporated into polymeric micelles exhibit favorable therapeutic advantages: enhanced drug solubility in water, prolonged circulation time by inhibiting phagocytic and renal clearance, and passive targeting to the tumor tissues by the enhanced permeability and retention effect.^2–5^ However, many drug delivery systems face strong limitations that potentially affect their further translation to clinical tests: the burst release in which a large fraction of adsorbed drug is rapidly released after administration and the inadequate drug loading which usually necessitates a high concentration of nanocarriers to obtain a noticeable therapeutic effect. These major obstacles may be overcome by applying the polymer prodrug approach, where the drug is covalently conjugated onto polymer backbones and side chains through labile linkers that can be cleaved in certain conditions. The drugs cannot be released before the degradable linking is cleaved. Therefore, polymer drug conjugates exhibit excellent storage stability, low systemic toxicity in circulation, and localized drug releases.^6,7^

Stimulus-responsive micelles formed from amphiphilic copolymers or polymer drug conjugates are promising materials for drug delivery and may lead to enhanced biological properties and efficiencies.^8–15^ Among the available stimuli, light is of particular interest because it can be highly localized in time and space.^16^ Photoactivable drug delivery systems are not only suitable for surface cancer treatment but also applicable to deep-seated cancer under endoscopic or optical fiber guidance, which is also used for photodynamic therapy.^17–20^ Recently, several research groups have demonstrated an irreversible response, involving the incorporation of the photocleavable units in the main chain of one of the blocks or in the hydrophobic cores conjugated with photocaged chemotherapeutic drugs, that induces the selective degradation of a specific micellar compartment. Among the many studied photolabile groups, *o*-nitrobenzyl (ONB) alcohol derivatives have received considerable attention in the area of synthetic polymer and polymer drug conjugates.^21–23^

**Scheme 1:**
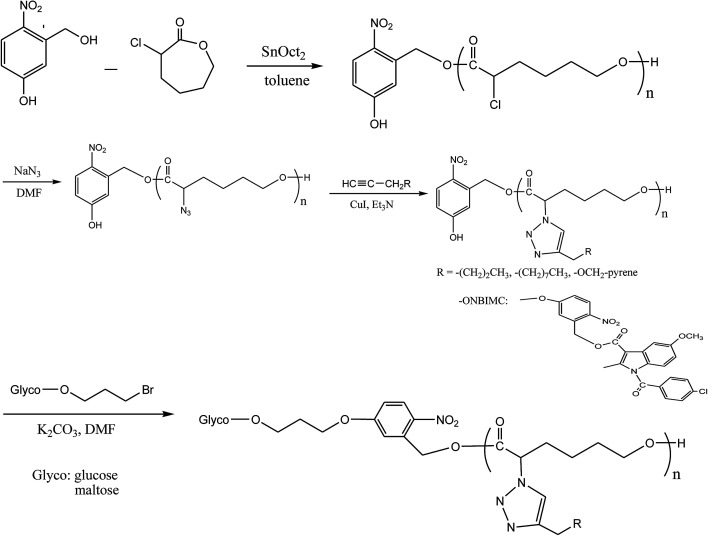
Synthesis photo-triggered Glyco-ONB-P(αN_3_CL-*g*-alkyne)_*n*_ and prodrug.

In this study, we present a synthetic platform, which permits *in situ* construction of various light-triggered drug delivery systems from amphiphilic glycose block functionalized poly(α-azo-ε-caprolactone) (Glyco-ONB-PαN_3_CL_*n*_) copolymers with photocleavable *ortho*-nitrobenzyl (ONB) units in the middle moiety and side chain, in which the drug molecules to be delivered can be loaded either physically (*i.e.*, the encapsulation approach) or chemically attached (*i.e.*, the prodrug approach). For the encapsulation approach, the ONB linked the hydrophilic block glycose and the hydrophobic block P(αN_3_CL)_*n*_ with the grafted alkyne, whereas the prodrugs were obtained by incorporating the linkers between the Glyco-ONB-PαN_3_CL_*n*_ and conjugating a drug molecule in the hydrophobic block. A potent nonsteroidal anti-inflammatory drug, indomethacin (IMC), is suitable drug model for studying micelle formation.^24^ The block–graft copolymers were synthesized through ring-opening polymerization (ROP), nucleophilic substitution, and “click” reactions (Scheme 1). The physicochemical and photodegradable properties of these micelles in the aqueous phase were examined through fluorescence spectroscopy, dynamic light scattering (DLS), and transmission electron microscopy (TEM). The phototriggered controlled release from the drug-loaded micelles and micelle drug conjugates in the physiological condition was reported. Finally, to demonstrate their potential as fluorescent probes in optical bioimaging, these DOX-encapsulated prodrug micelles were internalized in the human cervical cancer cell line HeLa and analyzed through fluorescence imaging and cytotoxicity study.

## Experimental section

### Materials

2-Chlorocyclohexanone (99%), 5-hydroxy-2-nitrobenzyl alcohol (97%), 1-hexyne, 1-decayne, sodium azide, pyrene (99%), dimethylamino-pyridine (DMAP, 99%), *N*,*N*′-dicyclohexyl-carbodiimide (DCC, >99%), IMC (99%), and Nile red (NR, 98%) were purchased from Aldrich Chemical Co. (Milwaukee, WI, USA). Moreover, *m*-chloroperoxybenzoic acid, α-d-pentaacetyl-glucopyranoside, d-(+)-maltose, and boron trifluoride diethyl etherate were purchased from Fluka Chemical Co. (Buchs, Switzerland). Stannous octoate (SnOct_2_, 95%) was purchased from Strem Chemical Inc. (Newburyport, MA, USA). 2-Chloro-ε-caprolactone (α-ClCL), 3-bromo-propyl-glucose, 3-bromo-propyl-maltose, 2-propargyoxymethyl were prepared according to previously described methods,^22,25^ but with modification. Doxorubicin hydrochloride (99%; Aldrich, Saint Louis, MO, USA) was deprotonated to obtain hydrophobic DOX as described previously.^26^*N*,*N*-Dimethyl formamide (DMF) and toluene were distilled under calcium hydride. Other high-pressure liquid chromatography (HPLC) grade solvents, such as tetrahydrofuran (THF), dimethylsulfoxide (DMSO), methanol, chloroform (CHCl_3_), ethyl acetate (EA), and *n*-hexane were purchased from Merck KGaA (Darmstadt, Germany). A Milli-Q Plus system (Waters, Milford, MA, USA) was used to obtain ultrapure water. Dulbecco's modified Eagle's medium (DMEM), trypsin/EDTA, 100× antibiotic antimycotic, and Hoechst 33342 nuclei dye were purchased from Gibco (Invitrogen Corp. Carlsbad, CA, USA). Fetal bovine serum (FBS) was obtained from Biological Industry (Kibbutz Beit Haemek, Israel). A CellTiter 96® AQueuous One Solution kit was obtained from Promega (Fitchburg, WI, USA) Amiloride, chlorpromazine, methyl-β-cyclodextrin, and nystatin were purchased from Sigma Aldrich (Saint Louis, MO, USA).

#### Prepare of 2-propargyoxymethyl pyrene (Ppyrene)

Pyrenemethanol (2 g, 8.61 mmol) was dissolved in toluene (40 mL) and added the KOH (9.64 g, 171.8 mmol). To the resulting suspension was slowly added propargyl bromide (25.6 g, 172.20 mmol) and stirred at 60 °C for 60 h. Then, the mixture was filtered and concentrated *in vacuo*. The residue was purified by flash column chromatography with hexane/EA (5 : 1) to give 2-propargyoxymethyl pyrene (1.51 g, 65% yield). ^1^H NMR (CDCl_3_, *δ*): 8.01–8.49 (m, 9H), 5.35 (s, 2H), 4.29 (d, 2H), 2.48 (d, 1H).

#### Prepare of 5-propargyoxy-2-nitrobenzyl indomethacinate (PONBIMC)

2-Nitro-5-propargyoxybenzyl alcohol^27^ (1.06 g, 5.10 mmol) and IMC (2.19 g, 6.12 mmol) was dissolved in THF (15 mL). Under N_2_ atmosphere, the DMAP (73 mg, 0.61 mmol) and DCC (3.2 g, 15.3 mmol) were added. The reaction mixture was stirred overnight at room temperature. Then, the mixture was filtered and concentrated *in vacuo*. The residue was purified by flash column chromatography with hexane/EA (5 : 1 to 2 : 1) to give 5-propargyoxy-2-nitrobenzyl indomethacinate (4.70 g, 86% yield). ^1^H NMR (CDCl_3_, *δ*): 8.18 (d, 1H), 7.79 (d, 2H). 7.49 (d, 2H), 7.0 (d, 1H), 6.98 (dd, 1H), 6.89 (d, 1H), 6.86 (d, 1H), 6.68 (d, 1H), 5.59 (s, 2H), 4.40 (s, 2H), 3.84 (s, 6H), 2.45 (s, 1H).

### Synthesis of HONB-P(αN_3_CL)_*n*_ polymers

**Fig. 1:**
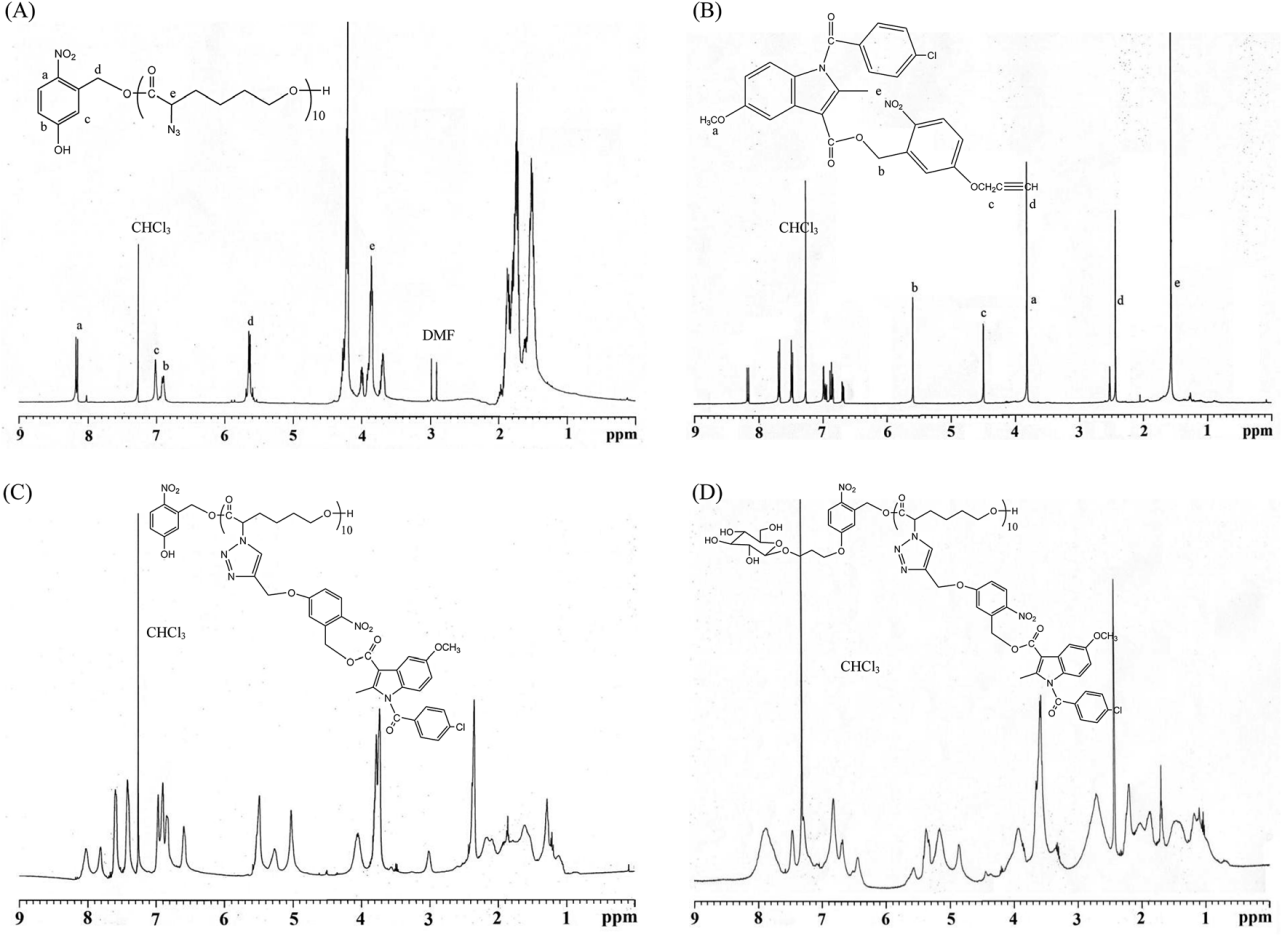
Representative ^1^H NMR spectroscopy: (A) HONB-P(αN_3_CL)_10_, (B) (4-propargoxy-2-nitro)benzyl indomethacinoate (PONBIMC), (C) HONB-P(αN_3_CL-*g*-PONBIMC)_10_, and (D) Gluco-ONB-P(αN_3_CL-*g*-PONBIMC)_10_.

**Fig. 2:**
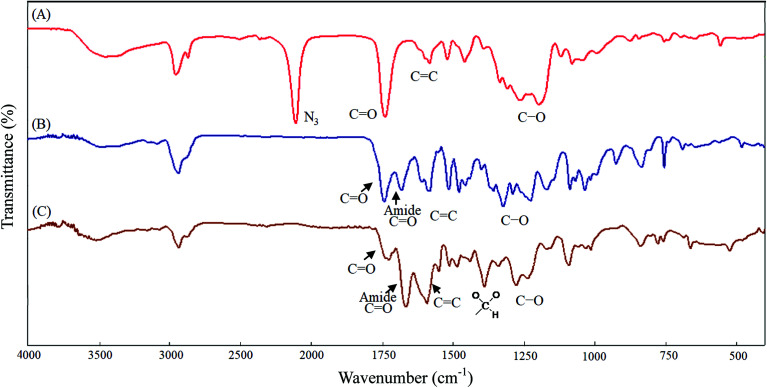
IR spectra of (A) HONB-P(αN_3_CL)_10_, (B) HONB-P(αN_3_CL-*g*-PONBIMC)_10_, (C) Gluco-ONB-P(αN_3_CL-*g*-PONBIMC)_10_.

All glassware was dried in an oven and handled under a dry nitrogen stream. 5-Hydroxy-2-nitrobenzyl alcohol (HONB, 275.1 mg, 1.63 mmol), as an initiator, and αClCL (2.41 g, 16.27 mmol) were introduced into a flask, heated under a dry nitrogen stream, and dissolved in 50 mL of toluene. Subsequently, SnOct_2_ (36 mg, 1.5 wt%, based on the weight of HONB and αClCL) was added to the flask. The flask was purged with nitrogen and refluxed for 8 h, and the solution was vacuum-concentrated under reduced pressure. The resulting product (HONB-P(αClCL)_10_) was dissolved in CHCl_3_, and precipitated into excess *n*-hexane/diethyl ether (5 : 1 v/v) with stirring. The purified polymer was dried *in vacuo* at 50 °C for 24 h and analyzed subsequently. Yield: 85%. ^1^H NMR (400 MHz, CDCl_3_) *δ* ppm: 8.18 (d, Ar*H*), 7.04 (d, Ar*H*), 6.88 (dd, Ar*H*), 5.60 (s, benzyl C*H*_2_–), 4.25 (t, –C_α_*H*Cl–), 4.21 (t, –C*H*_2_O–), 1.91–2.15 (m, –C_β_*H*_2_–), 1.74 (m, –C_δ_*H*_2_–), and 1.40–1.66 (m, –C_γ_*H*_2_–). Subsequently, the chloro was substituted by the azide. HONB-P(αClCL)_10_ (2.68 g, 1.28 mmol, molar mass = 2090 g mol^−1^) was dissolved in 10 mL of dry DMF, followed by the addition of excess NaN_3_ (1.01 g, 15.53 mmol). The mixture was stirred at room temperature for 24 h, the solvent was completely removed through rotary evaporation under reduced pressure. The crude product was subsequently dissolved in CH_2_Cl_2_, and the insoluble salt was removed through filtration. After concentration, the modified polymer was precipitated in excess cold diethyl ether. The copolymer HONB-P(αN_3_CL)_10_ was obtained at a 91% yield. Fig. 1A and 2A depict the representative ^1^H NMR and infrared (IR) spectra of HONB-P(αN_3_CL)_10_, respectively.

### Synthesis of HONB-P(αN_3_CL-*g*-alkyne)_*n*_ block-grafted polymer with pendant pyrene or IMC groups

HONB-P(αN_3_CL-*g*-alkyne)_*n*_ was prepared through copper-catalyzed azide–alkyne cycloaddition. HONB-P(αN_3_CL)_*n*_ (1.10 g) and various types of alkynes (such as 1-hexyne, 1-decayne, 2-propargyoxymethyl pyrene, and 5-propargyoxy-2-nitrobenzyl indomethacinate) were dissolved in THF (10 mL). Subsequently, CuI (0.01 eq.), and Et_3_N (0.1 eq.) were added under a nitrogen atmosphere. After one freeze pump thaw cycle, the click reaction was conducted at room temperature for 24 h and stopped by exposure to air. Copper ions were removed from the polymer solution by using a short alumina column. The polymer was purified by precipitating into an excess of cold diethyl ether and drying under reduced pressure. The yield of the copolymers was 83–92%. Fig. 1C and 2B depict the representative ^1^H NMR and IR spectra of HONB-P(αN_3_CL-*g*-PONBIMC)_10_, respectively.

### Synthesis of Glycol-ONB-P(αN_3_CL-*g*-alkyne)_*n*_ block-grafted polymers and prodrug

We used a typical procedure for coupling 3-bromopropyl-sugar with HONB-P(αN_3_CL-*g*-alkyne)_*n*_. First, a mixture of HONB-P(αN_3_CL-*g*-alkyne)_*n*_ (0.17 mmol) and potassium carbonate (71.6 mg, 0.52 mmol) was stirred in DMF (5 mL) for 1 h at 60 °C. 3-Bromopropyl d-glucopyranoside (63.2 mg, 0.21 mmol) in DMF (3 mL) was subsequently added, and the mixture was stirred at 60 °C for 24 h. DMF was removed under reduced pressure. The residue was dissolved in CHCl_3_, and precipitated into an excess of cold diethyl ether while stirring. The obtained solids were further purified through dialysis [cellulose membrane, molecular weight cutoff (MWCO): 3500 Da] against CHCl_3_ for 24 h. The purified polymers Glyco-ONB-P(αN_3_CL-*g*-alkyne)_*n*_ were dried *in vacuo* at 60 °C for 24 h to be obtained in 56–91% yield and analyzed. Fig. 1D and 2C depict the representative ^1^H NMR and IR spectra of Gluco-ONB-P(αN_3_CL-*g*-PONBIMC)_10_, respectively.

### Ultraviolet irradiation

Glyco-ONB-P(αN_3_CL-*g*-alkyne)_*n*_ (1.5 mg) in 1 mL of inhibitor-free THF or polymer micelles in phosphate buffer solution (PBS) (0.01 M, pH 7.4) were exposed to ultraviolet (UV) irradiation sourced from a UV light system (model PR-2000, Phnchum Co., Taiwan), equipped with a Hitachi FL8BL-B lamp (352 nm, 8 W × 8 W). To prevent UV absorption, the samples were placed in quartz cuvettes with a spot area of approximately 1 cm^2^ and irradiated at room temperature for an assigned duration. Radiation was applied vertically from the top of the cuvette.

### Preparation of polymeric micelles

Polymeric micelles of Glyco-ONB-P(αN_3_CL-*g*-alkyne)_*n*_ were prepared through dialysis. Briefly, a solution of Glyco-ONB-P(αN_3_CL-*g*-alkyne)_*n*_ (30 mg) in 5 mL of DMF was transferred in a dialysis bag with a MWCO of 3500 Da. The solution was dialyzed against deionized water at an ambient temperature for 24 h. The water was replaced at 2 h intervals.

### Determination of drug loading content and drug entrapment efficiency

Glyco-ONB-P(αN_3_CL-*g*-alkyne)_*n*_ [50-fold critical micelle concentration (CMC) value] was dissolved in 6 mL of methylene chloride using the oil-in-water evaporation technique. IMC, the anti-inflammatory drug, served as a model drug and was added to the polymer at a 1 : 1 weight ratio. The solution was added dropwise to 150 mL of distilled water containing 1 wt% polyvinyl alcohol and stirred vigorously. Polyvinyl alcohol acted as a surfactant and reduced micellar aggregation. The mixture was sonicated for 1 h at an ambient temperature to reduce the droplet size. The resulting emulsion was stirred at an ambient temperature overnight to evaporate the methylene chloride. The unloaded IMC residue was removed through filtration using a Teflon filter (Whatman) with an average pore size of 0.45 μm. The micelles were obtained through vacuum drying. Later, a weighed amount of micelles was disrupted by adding a 10-fold excess volume of DMF. The drug content was assayed spectrophotometrically at 320 nm using a diode array UV-visible spectrophotometer. The following equations were used to calculate the drug loading content (DLC) and drug entrapment efficiency (DEE):

1 DLC (%) = (weight of the drug in micelles/weight of micelles) × 100

2 DEE (%) = (weight of the drug in micelles/weight of the drug provided initially) × 100

### Analysis of *in vitro* drug release

Appropriate amounts of IMC-incorporated or conjugated micelles (110.2 mg) were weighed and suspended in 10 mL of PBS (0.01 M, pH 7.4). The micellar solution was transferred to a dialysis membrane bag (MWCO = 3500 Da), and the bag was placed in 50 mL of a PBS release medium. The medium was exposed to UV irradiation at 37 °C. At predetermined intervals, 3 mL aliquots of the aqueous solution were withdrawn from the release medium, and an identical volume of the fresh buffer solution was added. The concentration of the released IMC was determined using a UV-visible spectrophotometer at a wavelength of 320 nm. The rate of controlled drug release was measured on the basis of the cumulatively released weight of the drug by using the calibration curve for the drug.

### Carbohydrate–lectin binding recognition

The lectin recognition activity of the Glyco-ONB-P(αN_3_CL-*g*-alkyne)_*n*_ solution was analyzed by assessing the change in turbidity at 600 nm at room temperature. A 2 mg mL^−1^sample of concanavalin A (Con A) lectin was prepared in PBS (0.01 M, pH 7.4). Subsequently, 600 μL of the lectin solution was transferred into a cuvette, and the baseline measurement was recorded. A solution of 60 μL of Glyco-ONB-P(αN_3_CL-*g*-alkyne)_*n*_ at two concentrations (0.1 and 0.2 mg mL^−1^) in PBS was added to the cuvette containing the lectin solution. The solution in the cuvette was gently mixed using a pipette; thereafter, the absorbance was recorded immediately at 600 nm every 200 s. Control readings were obtained using lectin Con A and the PBS buffer solution without Glyco-ONB-P(αN_3_CL-*g*-alkyne)_*n*_ under the same experimental conditions.

### Determination of *in vitro* cellular viability

The CellTiter 96® AQueuous One Solution kit was used to determine cellular viability. The assay was conducted according to the manufacturer's instructions with minor modifications. HeLa cells were seeded in a 24 well plate (3 × 10^4^ cells per well) overnight and subsequently treated with various concentrations of polymers (or DMSO vehicles) added to the DMEM/F12 1 : 1 medium with 1% FBS in a humidified incubator at 37 °C, supplied with 5% carbon dioxide. After 48 h, the medium in each well was removed and replaced with 350 μL of warm PBS and 35 μL of CellTiter 96® AQueuous One Solution. The mixture was incubated at 37 °C for 4 h. After incubation, 110 μL of supernatant was transferred from each well to a 96 well plate and absorbance was measured at 485 nm using an enzyme-linked immunosorbent assay reader (Hidex, Turku, Finland). Each experiment was conducted in triplicate.

### Flow cytometric analysis of the uptake of doxorubicin-loaded micelles

HeLa cells were seeded in 35 mm dishes (1.5 × 10^5^ cells per dish) and cultured overnight. Subsequently, after UV irradiation or without treated DOX-loaded Gluco-ONB-P(αN_3_CL-*g*-PONBIMC)_10_ micelles and free DOX (447 ng mL^−1^) dissolved in DMEM/F12 1 : 1 medium with 1% FBS, were added and the cells were incubated for 1, 5, and 60 min. Next, the cells were trypsinized and fixed with 4% paraformaldehyde for 15 min prior to analysis. A BD FACS-Calibur flow cytometer (equipped with a 488 nm argon laser) and CellQuest software were used for the analysis. An FL2 channel captured the fluorescence of the DOX. Each experiment was conducted in triplicate.

## Results and discussion

### Synthesis and characterization of the Glyco-ONB-P(αN_3_CL-*g*-alkyne)_*n*_ block-grafted copolymer

**Fig. 3:**
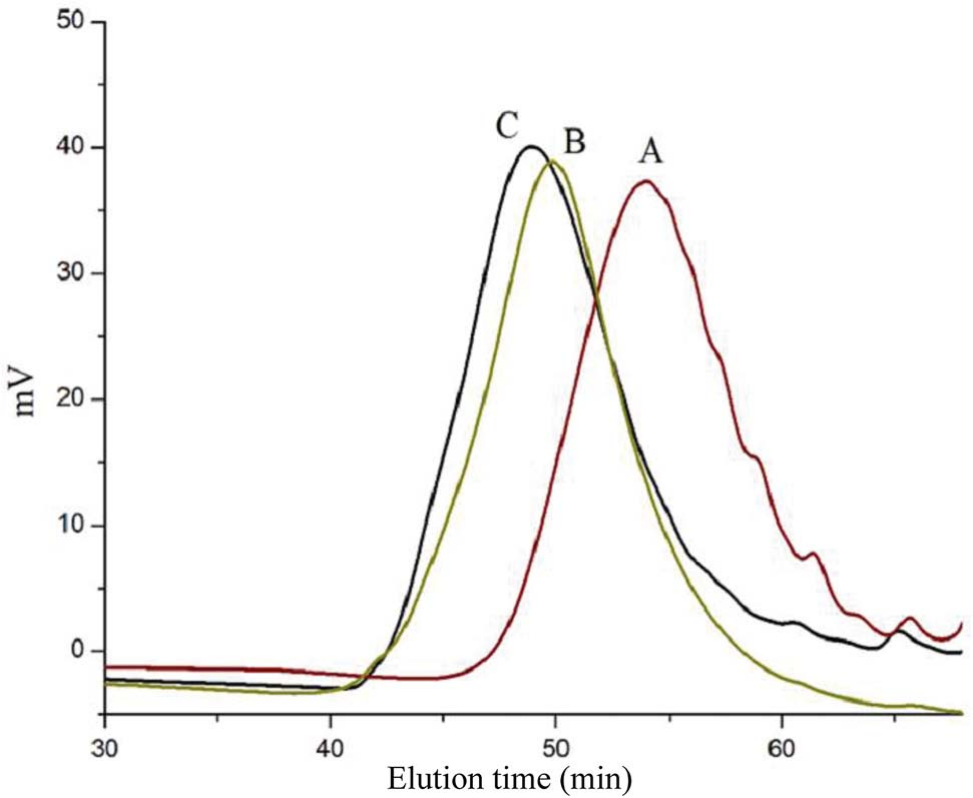
GPC curve of (A) HONB-P(αN_3_CL)_10_, (B) HONB-P(αN_3_CL-*g*-PONBIMC)_10_, and (C) Gluco-ONB-P(αN_3_CL-*g*-PONBIMC)_10_.

Scheme 1 depicts the strategy for synthesizing the biorecognizable and photocleavable Glyco-ONB-P(αN_3_CL-*g*-alkyne)_*n*_ copolymer. 5-Hydroxy-2-nitrobenzyl alcohol, a difunctional initiator, which contains two hydroxyl groups, namely benzyl and phenolic hydroxyl groups, was used as a photoresponsive molecule because of its chemical stability and rapid cleavage in response to near-UV irradiation (wavelength > 320 nm).^27,28^ Being more nucleophilic than the phenolic hydroxyl group, the benzyl hydroxyl group initiated the ROP of αClCL catalyzed by SnOct_2_. The HONB-P(αClCL)_*n*_ polymers with various P(αClCL)_*n*_ lengths were obtained by varying the molar ratio of HONB to αClCL. The degrees of polymerization (DP_n_) of αClCL can be calculated on the basis of the area ratios of peaks at 4.25 ppm (C*H*Cl of αClCL) to 5.80 (benzyl protons of HONB). Our calculation result indicates that the average DP of αClCL is approximately 10, and the number-average molecular weight (*M*_n_) of the polymer is approximately 2 kDa. Subsequently, HONB-P(αN_3_CL)_10_ was prepared by a reaction of HONB-P(αClCL)_10_ and sodium azide. After the reaction, a strong absorption at 2106 cm^−1^, representing the stretching vibration of azide groups, was observed in the Fourier transform IR (FTIR) spectrum of the polymer (Fig. 2A). The ^1^H NMR result confirms the conversion of the pendent chlorides to azides. The signal at 4.25 ppm corresponding to the methyne protons next to the chloride groups disappears completely; furthermore, a peak at 3.85 ppm corresponding to the methyne protons next to the azide groups can be observed. Fig. 3A depicts the gel permeation chromatography (GPC) curves of HONB-P(αN_3_CL)_10_ (*M*_n_ = 2150 g mol^−1^, *M*_w_/*M*_n_ = 1.19).

The pendant alkyne groups were grafted to HONB-P(αN_3_CL)_*n*_ backbone through copper-catalyzed azide–alkyne cycloaddition. The FTIR spectrum of HONB-P(αN_3_CL-*g*-PONBIMC)_10_ is shown in Fig. 2B. After the click reaction, the absorbance band at 2106 cm^−1^ disappeared completely, and new absorbance bands at 1680 cm^−1^, attributable to the amide carbonyl vibration of the IMC, were observed. The ^1^H NMR spectra of HONB-P(αN_3_CL-*g*-PONBIMC)_10_ is shown in Fig. 1C. New peaks at 5.30 ppm, corresponding to the methyne protons next to the triazole ring, and at 8.02 ppm, corresponding to the vinylic protons of triazole, were observed. Other peaks, including the signals at 7.81, 7.59, 5.61, 4.51, and 3.81 ppm of the IMC, were observed. The GPC trace of HONB-P(αN_3_CL-*g*-PONBIMC)_10_ is shown in Fig. 3B (*M*_n_ = 5490 g mol^−1^, *M*_w_/*M*_n_ = 1.43), with unimodal distribution and a shift towards the higher molecular weight region compared with the original HONB-P(αN_3_CL)_10_.

Finally, the phenolic hydroxyl group of the resulting HONB-P(αN_3_CL-*g*-alkyne)_*n*_ polymer was etherified using Glyco-(CH_2_)_3_Br (*e.g.*, Gluco-(CH_2_)_3_Br and Malto-(CH_2_)_3_Br) through nucleophilic substitution in DMF at 60 °C to yield the Glyco-ONB-P(αN_3_CL-*g*-alkyne)_*n*_ polymer. Table 1 presents the coupling results. The *M*_n,GPC_ of the block copolymers with different compositions ranged from 6100 to 10 450 mg mol^−1^, and the polydispersity index (*M*_w_/*M*_n_) ranged from 1.08 to 1.79. The ^1^H NMR and FTIR results confirmed the effective coupling of Gluco-(CH_2_)_3_Br to yield Gluco-ONB-P(αN_3_CL-*g*-PONBIMC)_10_. The representative ^1^H NMR spectrum is depicted in Fig. 1D. The resonance peaks were assigned to the corresponding hydrogen atoms of the Gluco-blocks at *δ* 5.05–5.39, and 3.21–3.79 ppm. The presence of proton signals from a Gluco-unit suggested successful conjugation. The IR spectrum of Gluco-ONB-P(αN_3_CL-*g*-PONBIMC)_10_ indicated typical carbonyl absorption of an ester of ONB-P(αN_3_CL-*g*-PONBIMC)_10_ at 1720 cm^−1^, amide carbonyl absorption at 1680 cm^−1^, hemiacetal C–O absorption at 1400 cm^−1^, and alcohol C–O absorption at 1250 cm^−1^ for glucose (Fig. 2C). Fig. 3C depicts the representative GPC curves of Gluco-ONB-P(αN_3_CL-*g*-PONBIMC)_10_ (*M*_n_ = 6280 g mol^−1^, *M*_w_/*M*_n_ = 1.40) and depicts a unimodal distribution with a shift in the peak towards the higher molecular weight region, compared with that of the HONB-P(αN_3_CL-*g*-PONBIMC)_10_.

**Table 1:** Results of the coupling of HONB-P(αN_3_CL-*g*-alkyne/or drug)_*n*_ with Glyco(CH_2_)_3_Br

Polymer^a^	*M*_n,th_ b	*M*_n,NMR_ c	*M*_n,GPC_ d	*M*_w_/*M*_n_ (PDI)^d^	Isolated yield (%)
Gluco-ONB-P(αN_3_CL-*g*-Hexy)_18_	4658	7735	8580	1.29	91
Gluco-ONB-P(αN_3_CL-*g*-Decy)_17_	5370	8940	10450	1.79	69
Gluco-ONB-P(αN_3_CL-*g*-Ppyren)_12_	5489	6236	6100	1.17	56
Gluco-ONB-P(αN_3_CL-*g*-Ppyren_2_/-Hexy_24_)	6968	7080	7910	1.35	76
Malto-ONB-P(αN_3_CL-*g*-Ppyren_2_/-Hexy_24_)	7131	6948	7510	1.08	86
Gluco-ONB-P(αN_3_CL-*g*-PONBIMC)_10_	7400	6788	6280	1.40	83

a Abbreviations: Gluco = glucose; Malto = maltose; ONB = 5-hydroxy-2-nitrobenzyl alcohol; P(αN_3_CL) = poly(α-azo-ε-caprolactone); Hexy = 1-hexyne; Decy = 1-decayne; Ppyren = 2-propargyoxymethyl pyrene; PONBIMC = 5-propargoxy-2-nitro-benzyl indomethacinate.

b *M*_n,th_ = *M*_n,Glyco(CH_2_)_3__ + *M*_n,HONB-P(αN_3_CL-*g*-alkyne/or drug)_*n*__.

c Determined by ^1^H NMR.

d Determined by GPC.

### Glyco-ONB-P(αN_3_CL-*g*-alkyne)_*n*_ micelles

**Fig. 4:**
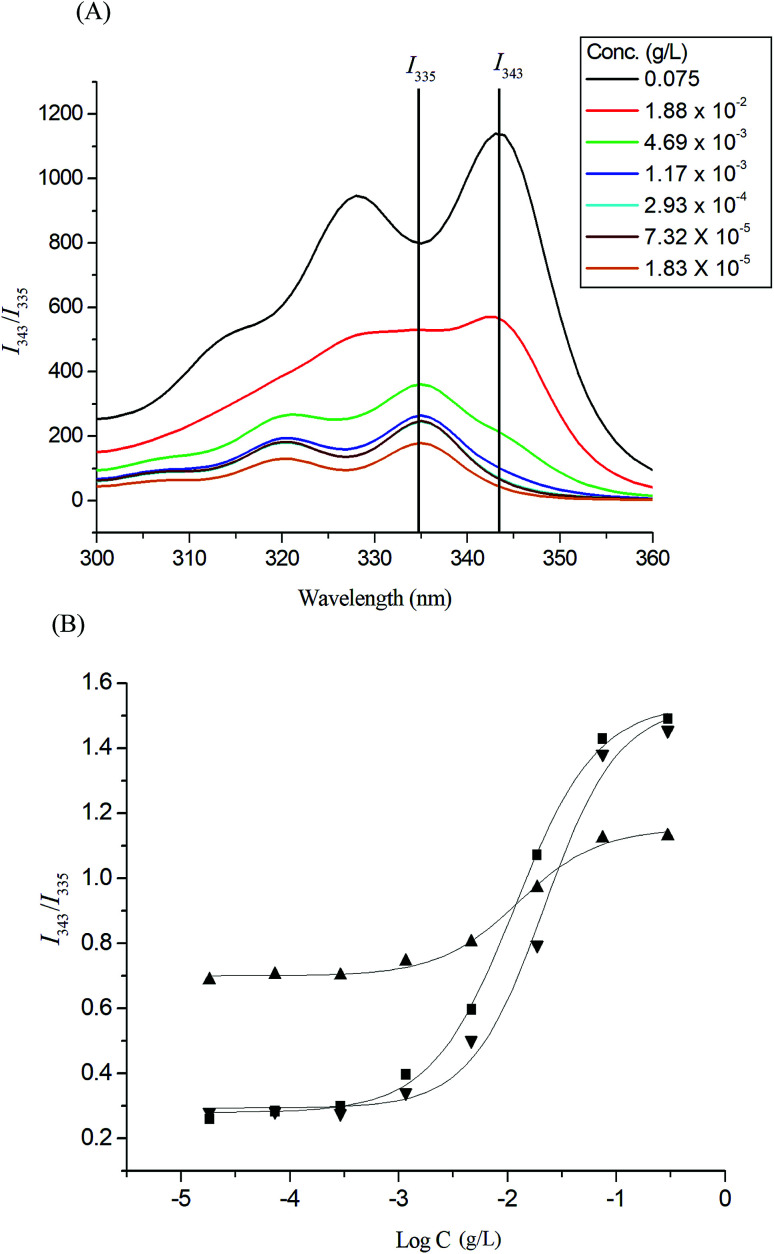
(A) Excitation spectra of pyrene Gluco-ONB-P(αN_3_CL-*g*-Ppyren)_12_ micelles monitored at *λ*_em_ = 390 nm with different concentrations. (B) Plot of the *I*_343_/*I*_335_ intensity ratio (from pyrene excitation spectra: pyrene concentration = 6.1 × 10^−7^ M) *versus* the logarithm of the concentration (log *C*) of Glyco-ONB-P(αN_3_CL-*g*-alkyne)_*n*_: (■) Gluco-ONB-P(αN_3_CL-*g*-Ppyren)_12_, (▲) Gluco-ONB-P(αN_3_CL-*g*-Ppyren_2_/-Hexy_24_), (▼) Malto-ONB-P(αN_3_CL-*g*-Ppyren_2_/-Hexy_24_).

Pyrene molecules encapsulation into the Glyco-ONB-P(αN_3_CL-*g*-alkyne)_*n*_ polymers was investigated through fluorescence spectroscopy. The ratio of the intensity of the third emission peak to that of the first emission peak (*I*_343_/*I*_335_) was used to characterize the microenvironment of pyrene, with a larger *I*_343_/*I*_335_ indicating a more hydrophobic environment for the pyrene molecules. The fluorescence intensity of the excitation spectrum of pyrene increased with the concentration of the Gluco-ONB-P(αN_3_CL-*g*-Ppyren)_12_ polymer (Fig. 4A). The characteristic feature of the pyrene excitation spectrum, a red-shift of the (0, 0) band from 335 nm to 343 nm during partitioning into a micellar hydrophobic core, was used to determine the CMC values of Gluco-ONB-P(αN_3_CL-*g*-Ppyren)_12_. Fig. 4B presents the intensity ratios *I*_343_/*I*_335_ of the pyrene excitation spectra and the logarithmic values of the Glyco-ONB-P(αN_3_CL-*g*-alkyne)_*n*_ concentrations. The CMC value was determined according to the interaction between straight-line segments drawn through the points of the lowest polymer concentrations, which formed an almost horizontal line, and the points of the rapidly rising region of the plot. Table 2 lists the CMC values of various Glyco-ONB-P(αN_3_CL-*g*-alkyne)_*n*_ polymers. The Glyco-ONB-P(αN_3_CL-*g*-alkyne)_*n*_ polymers formed micelles in the aqueous phase, with the micellar CMC values ranging from 1.9 to 69.8 mg L^−1^. The Glyco-ONB-P(αN_3_CL-*g*-alkyne)_*n*_ polymers exhibited lower CMC values than those of the surfactant (*e.g.*, 2.3 g L^−1^ for sodium dodecyl sulfate in water), indicating thermodynamically favorable self-association in Glyco-ONB-P(αN_3_CL-*g*-alkyne)_*n*_ polymers. The CMC values decreased as the hydrophobicity of the hydrophobic segment increased. When the hydrophobic pyrene or IMC molecules were grafted onto the P(αN_3_CL)_*n*_, the CMC values decreased significantly.

**Table 2:** Properties of Glyco-ONB-P(αN_3_CL-*g*-alkyne)_*n*_ polymeric micelles and prodrug

Polymer	CMC (mg L^−1^)	Drug loading content^a^ (%)	Drug entrapment efficiency^a^ (%)	Micelle size^b^ (nm)					
				Blank	PD	Zeta potential (mv)	With IMC	PD	Zeta potential (mv)
Gluco-ONB-P(αN_3_CL-*g*-Hexy)_18_	69.8	8.21	16.43	86.1 ± 50.1	0.15	−20.7			
Gluco-ONB-P(αN_3_CL-*g*-Decy)_17_	65.6	11.36	22.72	157.2 ± 45.1	0.05	−31.9			
Gluco-ONB-P(αN_3_CL-*g*-Ppyren)_12_	1.9	48.07	96.14	115.2 ± 33.7	0.25	−38.2	149.8 ± 40.2	0.12	−31.9
Gluco-ONB-P(αN_3_CL-*g*-Ppyren_2_/-Hexy_24_)	2.1	23.11	46.23	156.9 ± 38.3	0.04	−41.5	189.0 ± 82.6	0.17	−32.6
Malto-ONB-P(αN_3_CL-*g*-Ppyren_2_/-Hexy_24_)	4.4	17.88	35.76	105.0 ± 36.9	0.12	−28.4	106.0 ± 48.7	0.17	−27.3
Gluco-ONB-P(αN_3_CL-*g*-PONBIMC)_10_	18.2	52.71^c^	—	74.5 ± 33.0	0.16	−31.6			

a Feed weight ratio IMX/polymer = 1/1.

b Micelle size and particle size distribution (PD) determined by DLS.

c The IMC conjugation content (LC%) was calculated as LC% = *W*_IMC_/*W*_p_ × % where *W*_IMC_ and *W*_p_ refer to the weight of conjugated IMC drug within micelles and the weight of prodrug micelles, respectively.

**Fig. 5:**
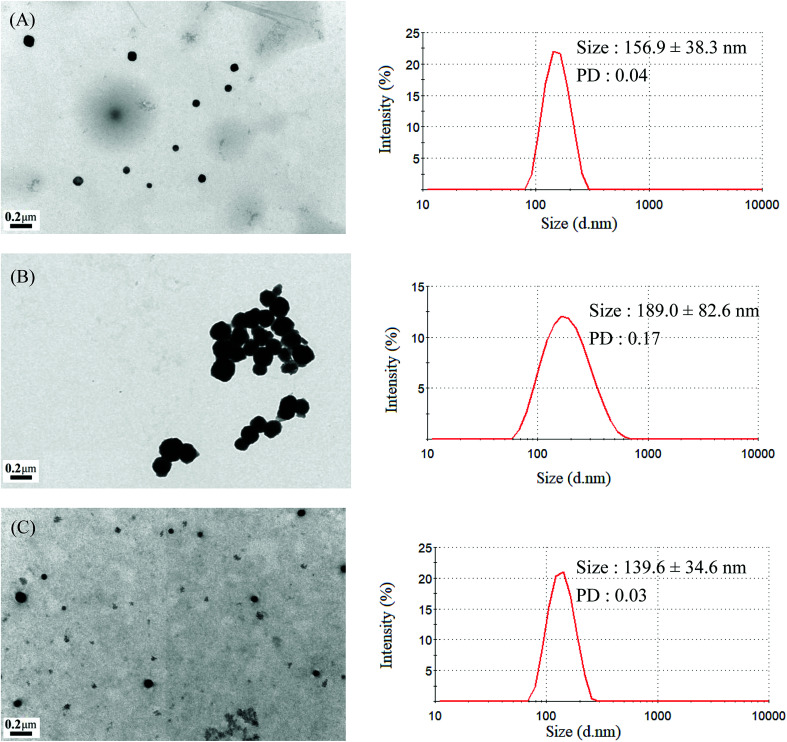
TEM and size distribution of Gluco-ONB-P(αN_3_CL-*g*-Ppyren_2_/-Hexy_24_) micelles: (A) blank, (B) IMC-loaded, and (C) after UV irradiation 6 h.

**Fig. 6:**
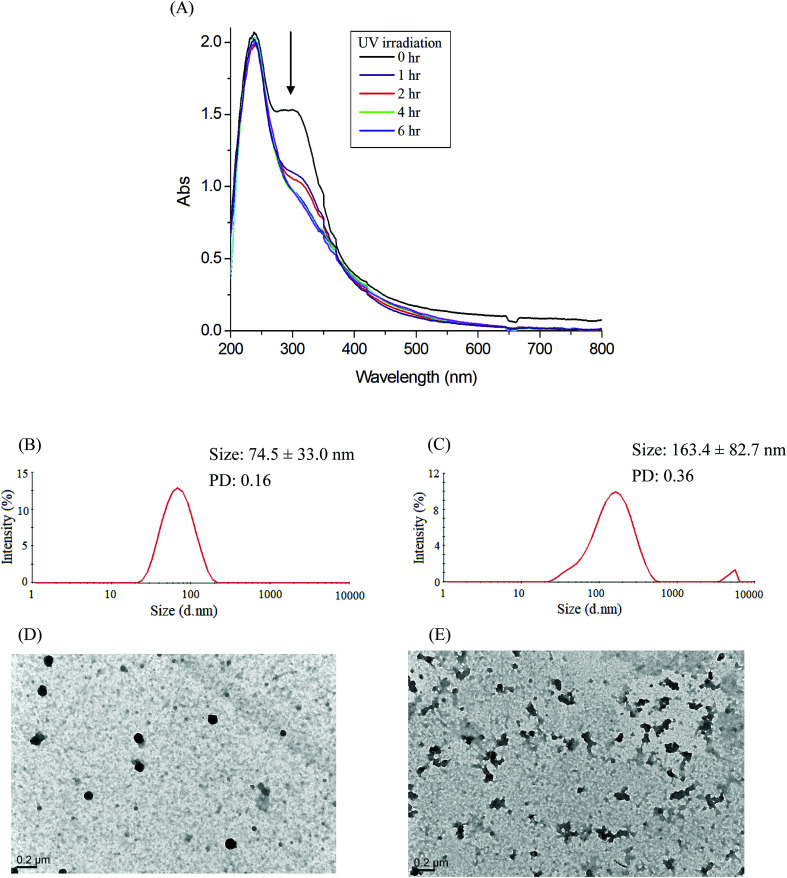
(A) Time-dependent UV-vis spectra changes of the IMC-conjugate Gluco-ONB-P(αN_3_CL-*g*-PONBIMC)_10_ micelles treatment under UV irradiation (352 nm, 8 W × 8 W), and DLS and TEM changes of Gluco-ONB-P(αN_3_CL-*g*-PONBIMC)_10_ micelles: without irradiation (B and D), and with irradiation for 6 h (C and E).

The mean hydrodynamic diameters of micelles ranged from 74.5 to 157.2 nm, with a narrow distribution, and demonstrated a particle size distribution (PD) of ≤0.25. The TEM and size distribution of Gluco-ONB-P(αN_3_CL-*g*-Ppyren_2_/-Hexy_24_) micelles are demonstrated in Fig. 5A. The spherical morphology was observed. When the drug was incorporated, the micellar size increased (Fig. 5B). The size of an IMC-incorporated Gluco-ONB-P(αN_3_CL-*g*-Ppyren_2_/-Hexy_24_) micelle was 189.0 nm larger than that of a blank micelle (156.9 nm). The increase in micelle size might be attributed to the increase of the hydrophobic core when the IMC is encapsulated into the copolymer, which is in agreement with the thermodynamic aggregation of the block copolymers (BCPs).^29^ However, the micelle size remained <200 nm for all formations. A suitable nanoparticle size (diameter: <200 nm) can reduce uptake in the reticulate endothelial system, minimize renal excretion, and increase micelle-encapsulated drug accumulation in tumors through increased permeability and retention.^30^ The average diameters measured through DLS were larger than those estimated using through TEM images. This is because DLS reveals the average dimensions of the micelles in aqueous solution, whereas TEM reveals the actual core dimensions of the micelles in a dry state. The TEM and PD of the prodrug Gluco-ONB-P(αN_3_CL-*g*-PONBIMC)_10_ micelles are presented in Fig. 6B and D, respectively.

**Fig. 7:**
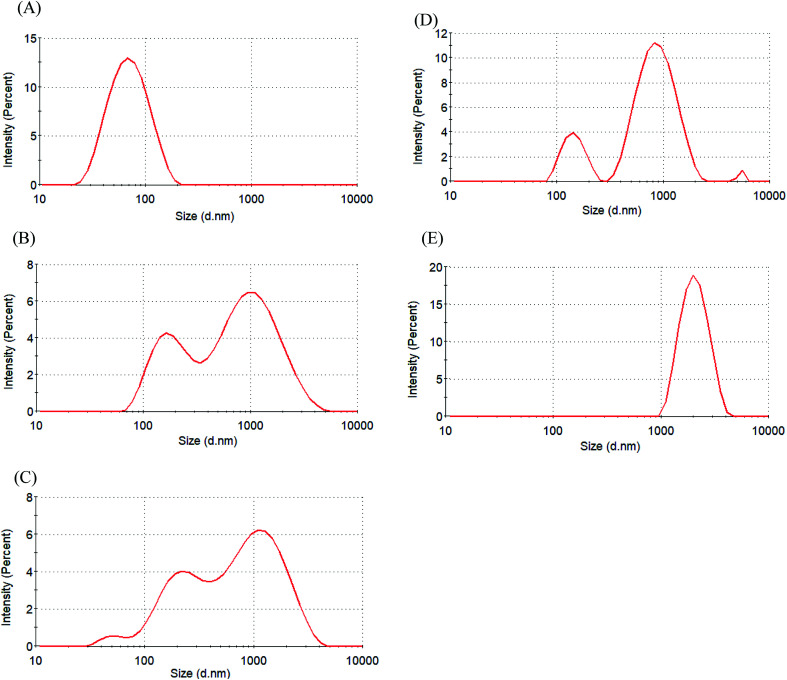
Size distribution of Gluco-ONB-P(αN_3_CL-*g*-PONBIMC)_10_ micelle in the presence of bovine serum albumin (10 wt%) for different time intervals: (A) blank, (B) 30 min, (C) 4 h, (D) 6 d, and (E) 14 d.

The zeta potential was used to assess micellar stability. When the absolute value of the zeta potential of a micelle was ≥30 mV, the micelle was stable.^31^Table 2 indicates that the zeta potential of Glyco-ONB-P(αN_3_CL-*g*-alkyne)_*n*_ micelles is in the range of −20.7 mV to −41.5 mV. The micellar surfaces were negatively charged with comparable zeta potentials. IMC loading lowered the degree of negative charges on the micellar surface because the hydroxyl groups of glycose molecules were protonated by the carboxylic acid group in the IMC molecules. For example, the zeta potential of the IMC-incorporated Gluco-ONB-P(αN_3_CL-*g*-Ppyren_2_/-Hexy_24_) micelles decreased from −41.5 mV to −32.6 mV. A less negatively charged surface is favorable for preventing uptake by the phagocytic cells, resulting in faster clearance from blood.^32^ However, to check the stability of the prodrug Gluco-ONB-P(αN_3_CL-*g*-PONBIMC)_10_ micelles in the culture medium, we dispersed the prodrug micelles with 10% bovine serum albumin (BSA) and incubated them at 37 °C for 14 day. The stability was studied by DLS measurement and presented in Fig. 7. BSA-treated prodrug micelles exhibited drastic increase in size to about 190 nm and 1130 nm with bimodal curve within 30 min. As the incubated time up to 14 day, the size of micelle increased to 2170 nm. These results imply that the prodrug micelle is easily absorption with protein.

### Photocleavable behaviors of micelles

**Fig. 8:**
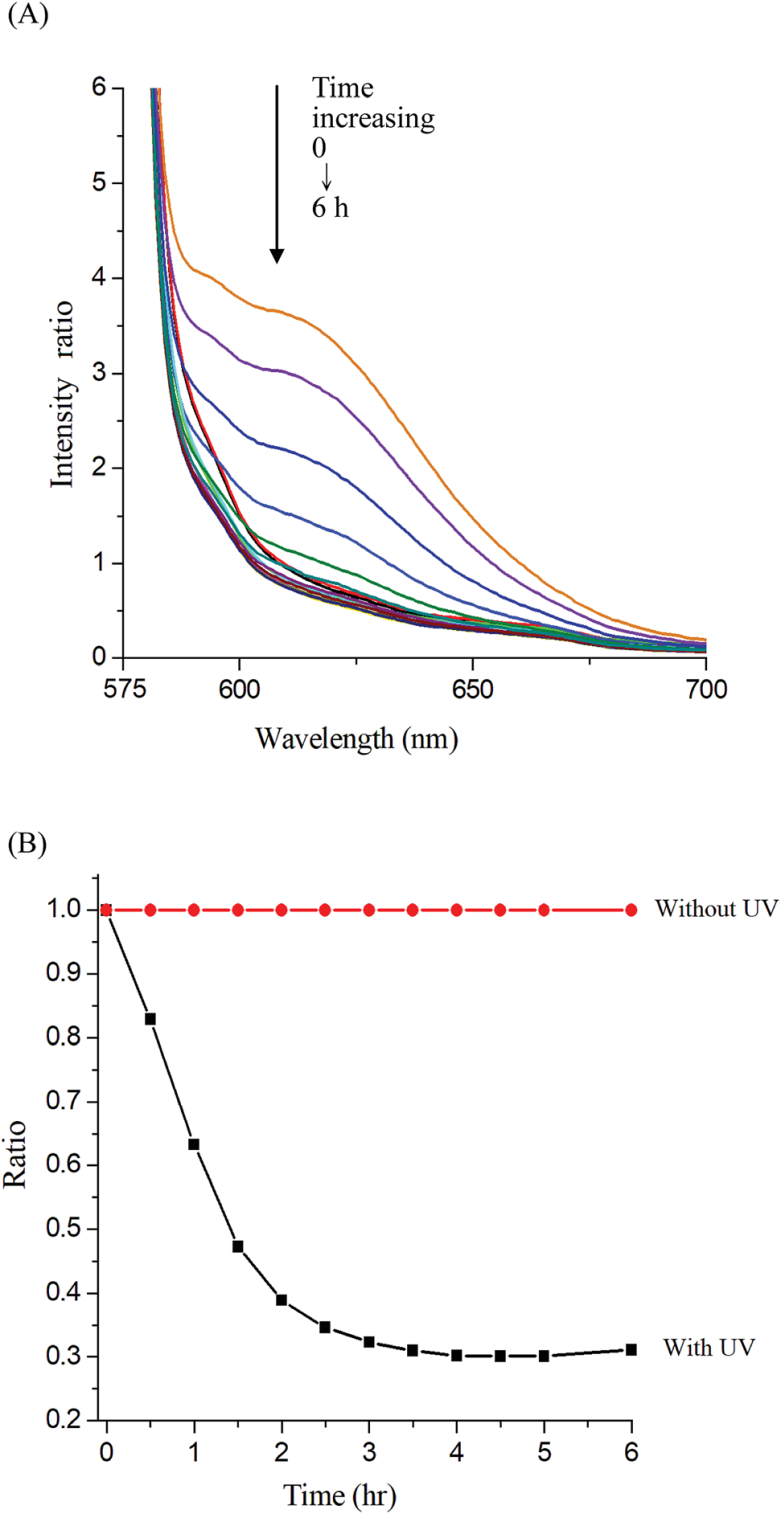
(A) Fluorescence spectra change of Nile red-loaded Gluco-ONB-P(αN_3_CL-*g*-Ppyren_2_/-Hexy_24_) micelle in PBS (0.01 M, pH 7.4) in the presence of UV irradiation (352 nm) at 25 °C, (B) normalized fluorescence emission intensity *vs.* time of irradiation.

Photo-degradation of micelles was assessed at various irradiation times by monitoring changes in NR fluorescence.^33^ The reduction in the intensity of NR fluorescence at 610 nm was recorded. After dissolving the NR and Gluco-ONB-P(αN_3_CL-*g*-Ppyren_2_/-Hexy_24_) in THF (0.5 mg mL^−1^, NR: polymer ratio = 1 : 3), water was added to induce micelle formation and concomitant NR encapsulation by the micelle core. THF was subsequently removed through evaporation, and nonsolubilized NR was micro-filtered (0.2 μm pore filter). The final micellar concentration was adjusted to 0.2 mg mL^−1^. Fig. 8A depicts the fluorescence emission spectra of NR incorporated in the Gluco-ONB-P(αN_3_CL-*g*-Ppyren_2_/-Hexy_24_) micelles before and after UV irradiation at various time intervals. Fig. 8B plots normalized fluorescence against time; through UV irradiation of the solution, the emission intensity was reduced to 30% after 6 h of irradiation. The micelles exhibited photolabile properties in response to light activation. The TEM and PD of Gluco-ONB-P(αN_3_CL-*g*-Ppyren_2_/-Hexy_24_) micelles after 6 h UV irradiation are presented in Fig. 5C. The results indicated that the destruction of a micelle was not complete because of only one ONB moiety and the steric hindrance of polymer. The photo-degradability of the formed prodrug micelles was subsequently studied. The TEM and size distribution of Gluco-ONB-P(αN_3_CL-*g*-PONBIMC)_10_ micelles after 6 h UV irradiation are demonstrated in Fig. 6C and E, respectively. Before irradiation, micelles morphologies were uniform. However, after UV irradiation, disintegrated micelles with aggregation were observed, indicating that irradiation for a specific duration induced changes in the assembly state. When expose to UV light, the micelle size of Gluco-ONB-P(αN_3_CL-*g*-PONBIMC)_10_ increased from 74.5 (PD = 0.16) to 163.4 nm (PD = 0.36), with broader size distribution and larger aggregates with an average diameter > 750 nm. These aggregates are likely fragments of the hydrophobic backbone without Gluco, which arise from the backbone degradation and are insoluble in water. Compared with the photocleavage of Gluco-ONB-P(αN_3_CL-*g*-Ppyren_2_/-Hexy_24_) micelles, the Gluco-ONB-P(αN_3_CL-*g*-PONBIMC)_10_ micelles demonstrated easier photocleavage because they have greater numbers ONB moieties in the polymer.

### Evaluation of drug loading content, entrapment efficiency and *in vitro* release of IMC

The DLC and DEE of the polymeric micelles were determined through UV-visible absorption spectroscopy of IMC. IMC, a widely used hydrophobic, nonsteroidal anti-inflammatory drug, was used as a model drug to investigate drug loading in the hydrophobic core. The maximal absorption peak of IMC was proportional to its concentration at 320 nm. After releasing IMC and removing the polymer precipitate, the amount of loaded IMC was determined according to the absorbance at 320 nm. Table 2 lists the calculated drug loading content and entrapment efficiency. At a constant feed weight ratio (*W*_IMC_/*W*_copolymer_ = 1 : 1), the DLC and DEE ranged from 8.21% to 48.07% and 16.43% to 96.14%, respectively, for the Glyco-ONB-P(αN_3_CL-*g*-alkyne)_*n*_ series of polymers. The DLC and DEE increased with the hydrophobicity of the hydrophobic segment. When the longer hydrophobic alkyne or pyrene was grafted onto the hydrophobic block, which exhibits stronger interactions with guest molecules, higher DLC and DEE can therefore be achieved. For the Gluco-ONB-P(αN_3_CL-*g*-PONBIMC)_10_ prodrug, the DLC was calculated approximately 52.71%. Compare with the simple physical encapsulation system, the DLC of the prodrug is high.

**Fig. 9:**
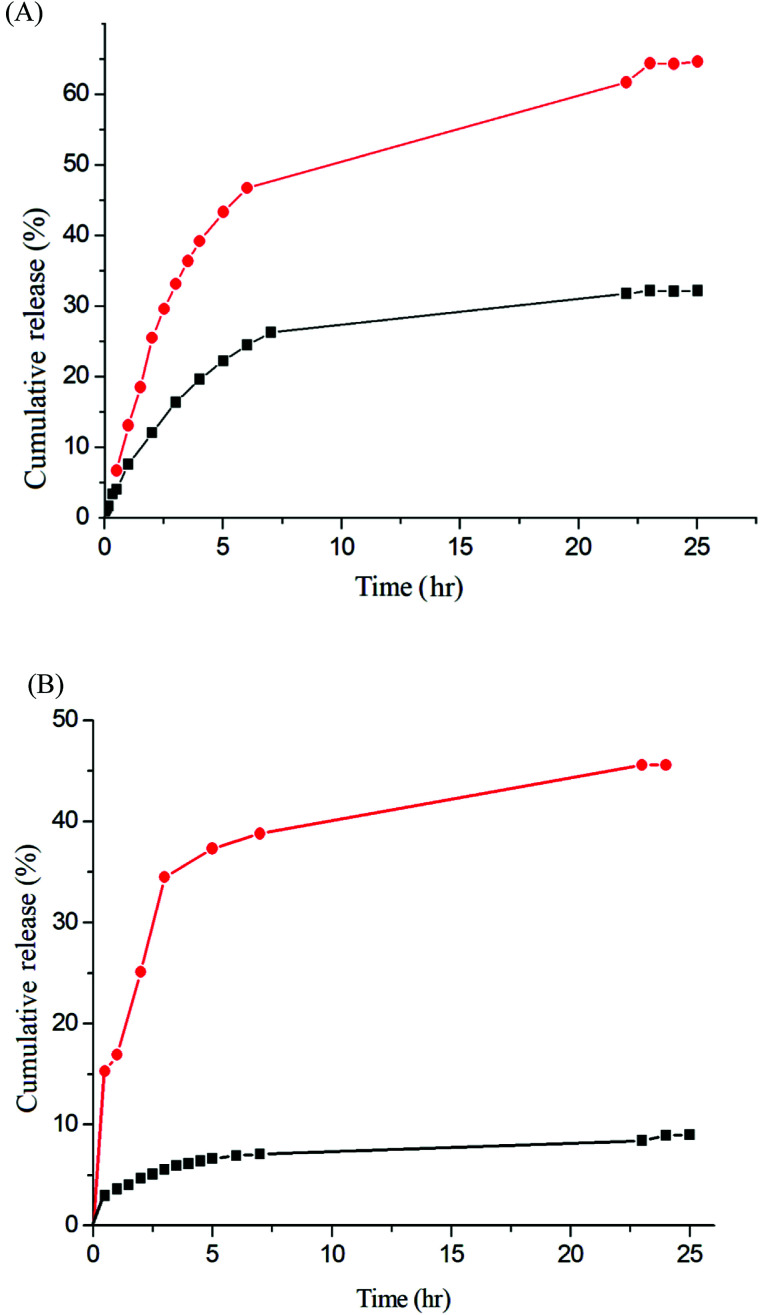
IMC release (A) the IMC-loaded micelle of Gluco-ONB-P(αN_3_CL-*g*-Ppyren_2_/-Hexy_24_) in the presence of UV irradiation (●) and without irradiation (■), (B) the IMC-conjugate micelle of Gluco-ONB-P(αN_3_CL-*g*-PONBIMC)_10_ in the presence of UV irradiation (●) and without irradiation (■) in PBS (0.01 M, pH 7.4) at 37 °C.

The release rate was monitored by determining the concentration of the total amount of released drug. Fig. 9 depicts the release profiles of IMC from the IMC-loaded micelles of Gluco-ONB-P(αN_3_CL-*g*-Ppyren_2_/-Hexy_24_) and IMC-conjugated micelles of Gluco-ONB-P(αN_3_CL-*g*-PONBIMC)_10_ prodrug. A biphasic release profile was observed, in which a rapid release stage was followed by a sustained release phase. Compared with the released rates in the absence of UV irradiation, the release rate of IMC was faster during UV irradiation at 37 °C, with approximately 45% of the encapsulated IMC released in a sustained manner during the first 5 h. After irradiation for 25 h, approximately 65% of the IMC was released, whereas only 26% release was exhibited without UV irradiation. For the IMC-conjugated Gluco-ONB-P(αN_3_CL-*g*-PONBIMC)_10_ prodrug system, the release rate of IMC was slower than the IMC-loading micelles of Gluco-ONB-P(αN_3_CL-*g*-Ppyren_2_/-Hexy_24_). Approximately 45% of the IMC was released under UV irradiation for 25 h; only 8% release was observed without irradiation over the same period (Fig. 9B). This can be ascribed to the covalent linkage nature between IMC and polymer backbones within micellar cores. Therefore, those covalent linkages can be cleaved with UV irradiation.

### Carbohydrate–lectin binding recognition

**Fig. 10:**
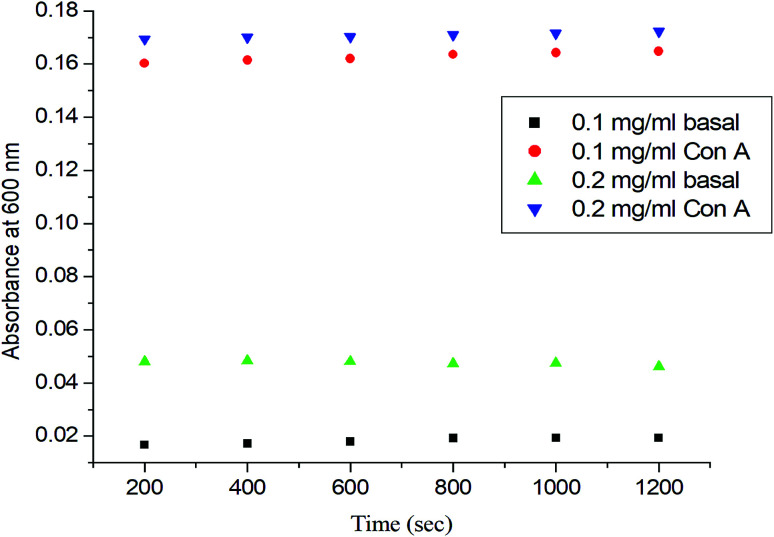
The absorbance (450 nm) of the Gluco-ONB-P(αN_3_CL-*g*-PONBIMC)_10_ solution upon reaction with lectin Con A (2 mg mL^−1^) in PBS buffer: concentration 0.2 mg mL^−1^ with lectin Con A (▼) or without lectin Con A (▲), concentration 0.1 mg mL^−1^ with lectin Con A (●) or without lectin Con A (■).

To determine the role of the synthetic glycopolymer, Glyco-ONB-P(αN_3_CL-*g*-alkyne)_*n*_, in drug targeting, the ability of the synthesized Gluco-ONB-P(αN_3_CL-*g*-PONBIMC)_10_ polymer to interact with the biological system was assessed. Carbohydrate is essential in several biological recognition events mediated by specific carbohydrate–lectin interactions. Although the exact mechanism of this interaction remains unknown, numerous studies have demonstrated that the mechanism is highly specific and noncovalent.^34^ Thus, an *in vitro* evaluation of this specific binding event provides an initial test of the ability of a synthetic Gluco-ONB-P(αN_3_CL-*g*-PONBIMC)_10_ polymer to interact with the biological systems, such as in drug delivery development, tissue engineering, and biomedical material synthesis. These tests are typically conducted by mixing Gluco-ONB-P(αN_3_CL-*g*-PONBIMC)_10_ with a lectin that is selective for the sugar conjugated to the polymer.^35^ A positive result is obtained when a precipitate appears because of lectin aggregation; this can be measured as the reduction in the transparency of the solution. Con A is a specific lectin used for selective binding to glucosyl residues. Therefore, we investigated the change in absorbance of solutions of Gluco-ONB-P(αN_3_CL-*g*-PONBIMC)_10_ with Con A at 600 nm. Fig. 10 illustrates that the absorbance (*i.e.*, turbidity) increased with the concentration of Gluco-ONB-P(αN_3_CL-*g*-PONBIMC)_10_ because of the formation of larger aggregates. For a control reading, a PBS buffer solution without Con A was added to the glucose polymer. A marginal absorbance significantly lower than that for the sample containing Con A, was detected.^36^ These experiments confirmed that Gluco-ONB-P(αN_3_CL-*g*-PONBIMC)_10_ synthesized through the nucleophilic coupling of Gluco-(CH_2_)_3_Br to HONB-P(αN_3_CL-*g*-PONBIMC)_10_ entailed active biorecognition.

### *In vitro* cytotoxicities of the polymer

**Fig. 11:**
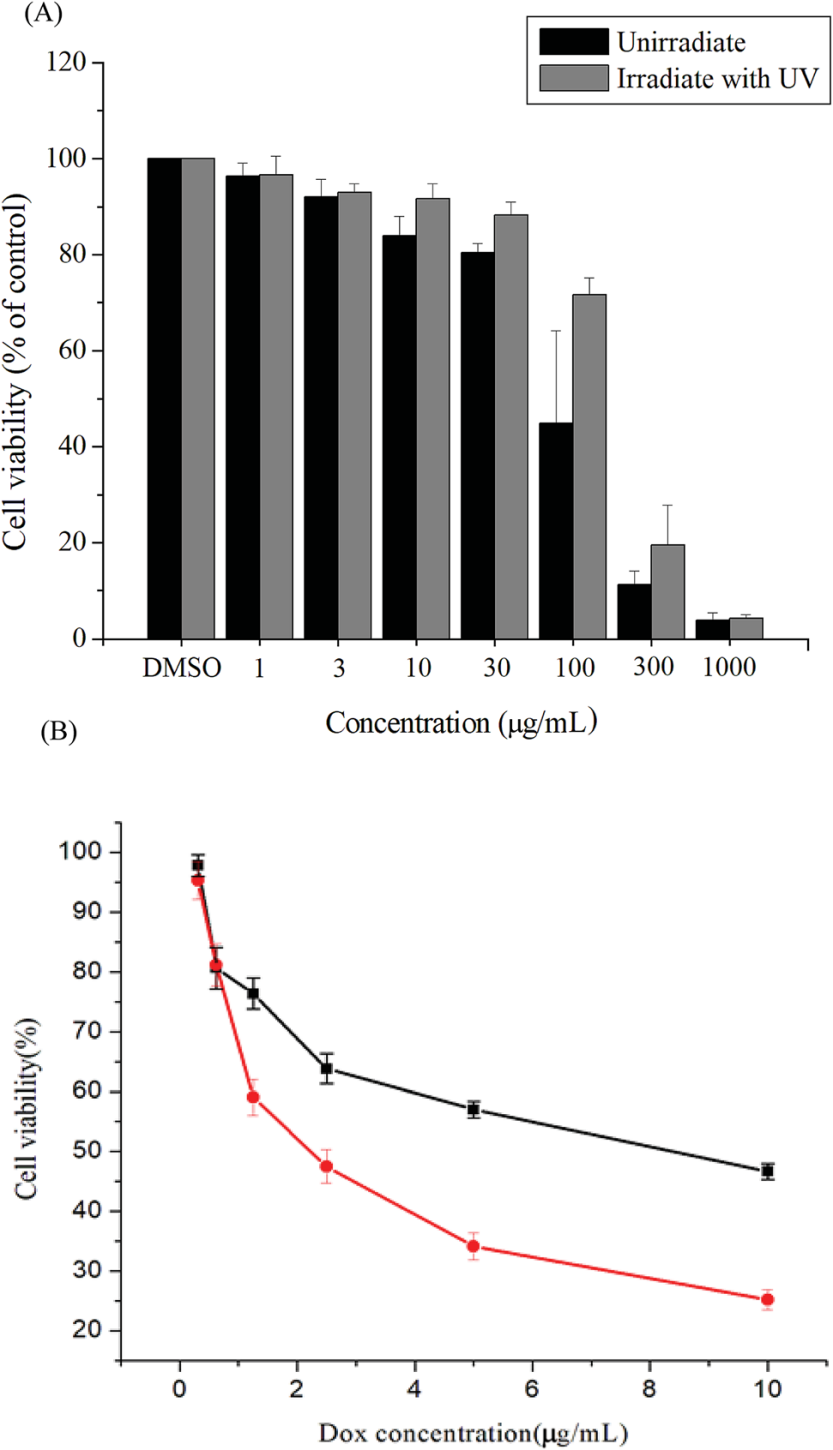
The cell viabilities of HeLa cells treated: (A) with various concentrations of Gluco-ONB-P(αN_3_CL-*g*-PONBIMC)_10_ before UV exposure and after 1 h of UV irradiation, (B) with DOX-loaded Gluco-ONB-P(αN_3_CL-*g*-PONBIMC)_10_ micelles (■), and free DOX (●) for 48 h. Data are shown as mean ± S.E. (*n* = 3).

Cytotoxicity is a crucial consideration in drug carrier design. *In vitro* cytotoxicities of the Gluco-ONB-P(αN_3_CL-*g*-PONBIMC)_10_ polymer with and without UV irradiation were evaluated through a 3-(4,5-dimethylthiazol-2-yl)-5-(3-carboxymethoxyphenyl)-2-(4-sulfophenyl)-2H-tetrazolium (MTS) assay of HeLa cells treated with various polymer concentrations. To eliminate the undesirable cytotoxic effects of the polymer with and without UV irradiation, the viability of cells loaded with increasing amounts of Gluco-ONB-P(αN_3_CL-*g*-PONBIMC)_10_ was assessed using a Promega CellTiter 96® AQueuous One Solution kit. HeLa cells were incubated with various concentrations of Gluco-ONB-P(αN_3_CL-*g*-PONBIMC)_10_ for 48 h followed by a reaction with the MTS reagent, which was bioreduced to formazan because of the esterase in living cells, enabling spectrophotometric analysis absorbance at 485 nm. Fig. 11A depicts the relative viability percentages of cells treated with various concentrations of Gluco-ONB-P(αN_3_CL-*g*-PONBIMC)_10_ before UV exposure and after 1 h of UV irradiation for 48 h. Cell viability was 80% higher than the control at a polymer concentration ranging from 1 to 30 μg mL^−1^. The results demonstrate that the Gluco-ONB-P(αN_3_CL-*g*-PONBIMC)_10_ solution before UV exposure and after 1 h of UV irradiation were slightly cytotoxic. In addition, Fig. 11B presents the *in vitro* cytotoxicities of the DOX-loaded micelles and free DOX at various DOX dosages (0.125–10 μg mL^−1^). The DOX-loaded Gluco-ONB-P(αN_3_CL-*g*-PONBIMC)_10_ micelles effectively in-habited HeLa cell proliferation with a half-maximal inhibitory concentration (IC_50_) of 8.8 μg mL^−1^. The DOX-loaded micelles possess higher IC_50_ than the free DOX (2.4 μg mL^−1^), which is observed in many polymeric system.^37^ This is likely caused by the longer time required for DOX to be released from micelles into tumor cells.

### Cellular uptake profile of doxorubicin-incorporated micelles

The cellular uptake of prodrug micelles by HeLa cells was investigated through flow cytometry and confocal laser scanning microscopy (CLSM). Amphiphilic drug-conjugated polymer can transport drugs through micelle formation. We investigated the drug-carrying ability of the polymeric Gluco-ONB-P(αN_3_CL-*g*-PONBIMC)_10_ micelles using DOX, a potent antitumor drug. The self-fluorescence characteristic of this drug facilitated the identification and quantification of drug-incorporated micelles that entered the cells.^38,39^

**Fig. 12:**
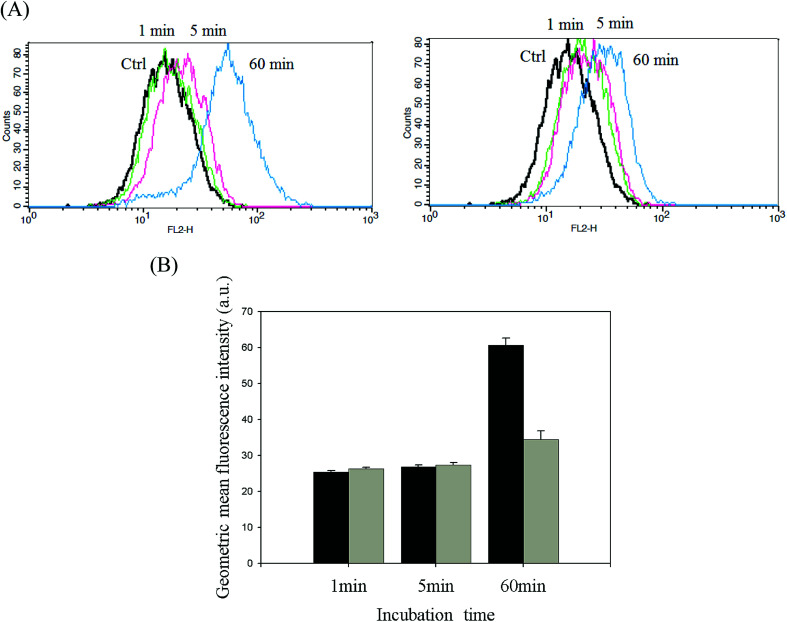
(A) Flow cytometric histogram profiles of HeLa cells treated with free DOX (left), and DOX-loaded Gluco-ONB-P(αN_3_CL-*g*-PONBIMC)_10_ (right) for 1, 5, and 60 min. Control groups were cells that did not receive any treatment, representing basal fluorescent levels, and (B) geometric mean fluorescence intensities of free DOX (black) and DOX-loaded micelles (gray). Data shown mean ± S. E. (*n* = 3).

**Fig. 13:**
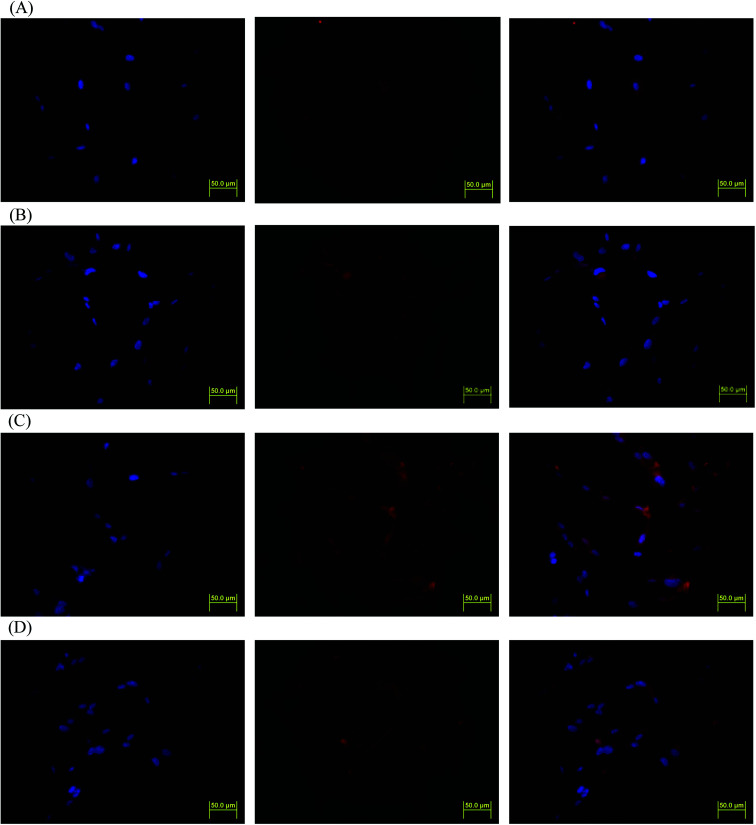
Fluorescent microscopic images of HeLa cells incubated with free DOX (254.7 ng mL^−1^) or DOX-loaded Gluco-ONB-P(αN_3_CL-*g*-PONBIMC)_10_ micelles for different time intervals: (A) free DOX, and (B) DOX-loaded micelles for 1 min; (C) free DOX, and (D) DOX-loaded micelles for 60 min. For each row, images for left to right show the cells with Hoechst 33342 nuclear staining, DOX fluorescence, and the merged image (scale bar 50 μm; brightness not proportional to fluorescence intensity).

DOX-incorporated Gluco-ONB-P(αN_3_CL-*g*-PONBIMC)_10_ micelles were prepared through dialysis, and the uptake of micelles and free DOX in equal concentration (458.2 ng mL^−1^) at 1, 5, and 60 min, respectively, were recorded through flow cytometry. In the first 5 min, DOX uptake in the cells incubated with DOX-incorporated micelles was similar to that in those treated with free DOX (Fig. 12A). However, DOX in its free-form enters and accumulates into cells faster than the DOX encapsulated in micelles at incubation time of up to 60 min. The geometric mean fluorescence intensity in the HeLa cells treated with DOX-incorporated Gluco-ONB-P(αN_3_CL-*g*-PONBIMC)_10_ was approximately 0.6-fold weaker than that in the HeLa cells treated with free DOX during the 60 min incubation time (Fig. 12B). Various fluorescence microscopic experiments were conducted to determine the intracellular distributions of DOX-incorporated Gluco-ONB-P(αN_3_CL-*g*-PONBIMC)_10_ micelles and free DOX following the cellular entry. Fig. 13 depicts the results of the nuclear stained Hoechst 33342 and DOX fluorescence as well as an overlay of the images (from left to right). These images indicate that free DOX and Gluco-ONB-P(αN_3_CL-*g*-PONBIMC)_10_-encapsulated DOX exhibited distinct temporal and spatial entry patterns. Free DOX accumulated in the cells at a substantially faster rate than did the Gluco-ONB-P(αN_3_CL-*g*-PONBIMC)_10_-encapsulated DOX, with a minimal fluorescence after 1 min of treatment, yielding visible fluorescence after 60 min of treatment (Fig. 13A and C). DOX fluorescence was predominantly concentrated in the cell nuclei, which could be an inherent tendency of the free DOX.^40^ By contrast, in the HeLa cells treated using DOX-incorporated Gluco-ONB-P(αN_3_CL-*g*-PONBIMC)_10_ for 1 or 60 min, DOX fluorescence was concentrated in the cytoplasm with little to no DOX visible in the nucleus (Fig. 13B and D). These differences are attributable to the free DOX entering the cells through passive diffusion, whereas the Gluco-ONB-P(αN_3_CL-*g*-PONBIMC)_10_ micelle-encapsulated DOX penetrated the plasma membrane through endocytosis.^41^ Although the uptake of the DOX-incorporated Gluco-ONB-P(αN_3_CL-*g*-PONBIMC)_10_ micelles was slow, the intensity of nuclear fluorescence in the HeLa cells treated with DOX-incorporated Gluco-ONB-P(αN_3_CL-*g*-PONBIMC)_10_ was marginally higher after 60 min than after 1 min (Fig. 13B and D). Entry into the nuclei by DOX confirmed that Gluco-ONB-P(αN_3_CL-*g*-PONBIMC)_10_ micelle-encapsulated DOX was released and successfully reached its pharmacological target. Low penetration of DOX-incorporated Gluco-ONB-P(αN_3_CL-*g*-PONBIMC)_10_ micelles into the extravascular tumor tissues may be attributed primarily to bulky micelle systems being substantial hindered from penetrating into tumor cells.^42^

**Fig. 14:**
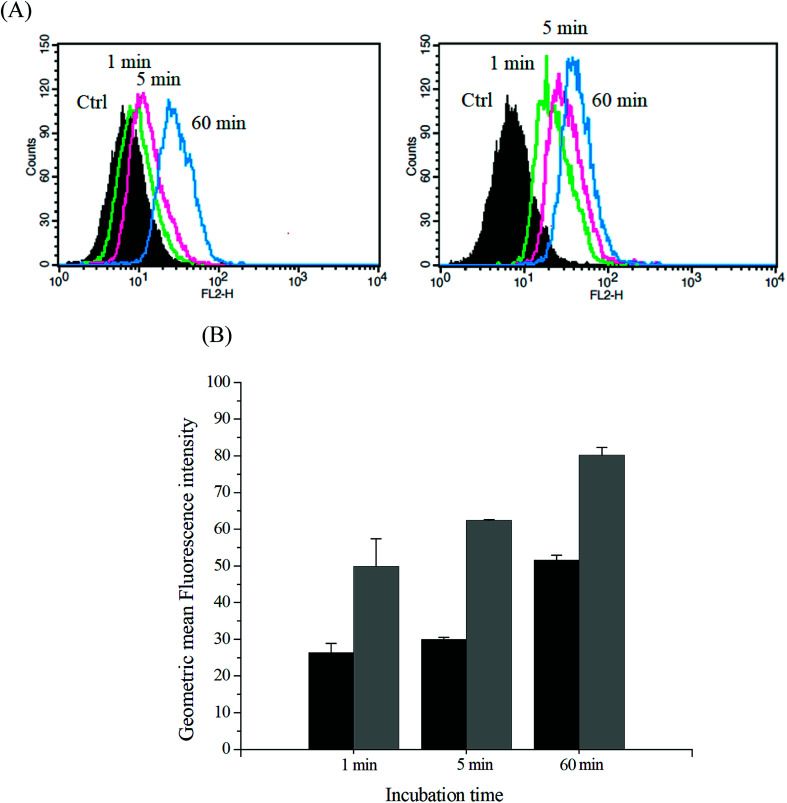
(A) Flow cytometric histogram profiles of HeLa cells treated with free DOX (left), and after 5 min UV irradiation DOX-loaded Gluco-ONB-P(αN_3_CL-*g*-PONBIMC)_10_ micelles (right) for 1, 5, and 60 min. Control groups were cells that did not receive any treatment, representing basal fluorescent levels, and (B) geometric mean fluorescence intensities of free DOX (black) and after 5 min UV irradiation DOX-loaded micelles (gray). Data shown mean ± S. E. (*n* = 3).

**Fig. 15:**
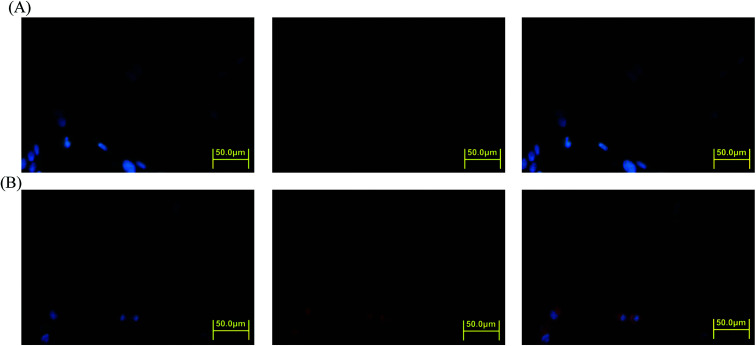
Fluorescent microscopic images of HeLa cells incubated with free DOX (254.7 ng mL^−1^) or after 5 min UV irradiation DOX-loaded Gluco-ONB-P(αN_3_CL-*g*-PONBIMC)_10_ micelles for 60 min: (A) free DOX, and (B) after UV irradiation DOX-loaded micelles. For each row, images for left to right show the cells with Hoechst 33342 nuclear staining, DOX fluorescence, and the merged image (scale bar 50 μm; brightness not proportional to fluorescence intensity).

The intracellular DOX release from DOX-incorporated Gluco-ONB-P(αN_3_CL-*g*-PONBIMC)_10_ under UV irradiation was also confirmed by flow cytometry and CLSM studies. As observed from Fig. 14 and 15, HeLa cells incubated with DOX-incorporated Gluco-ONB-P(αN_3_CL-*g*-PONBIMC)_10_ micelles after UV irradiation for 5 min displayed stronger intracellular DOX fluorescence intensity as compared to the free DOX. The DOX-incorporated micelles after UV irradiation for 5 min enters and accumulates into cells faster than the free-form DOX during the incubated time (Fig. 14A). The geometric mean fluorescence intensity in the HeLa cells treated with DOX-incorporated Gluco-ONB-P(αN_3_CL-*g*-PONBIMC)_10_ under UV irradiation was approximately 1.6-fold stronger than that in the HeLa cells treated with free DOX at incubation time of up to 60 min (Fig. 14B). Fig. 15 depicts the results of the nuclear stained Hoechst 33342 and DOX fluorescence as well as an overlay of the images (from left to right). These images indicate that the HeLa cells treated using DOX-incorporated micelles after UV irradiation for 60 min, DOX fluorescence was major concentrated in the cytoplasm. These results displayed that the cellular uptake of prodrug micelles under UV irradiation enters and accumulates into cells faster than without UV irradiation.^43^

## Conclusions

A family of phototriggered amphiphilic block-grafted copolymers and conjugated IMC in the hydrophobic segment prodrug were synthesized. The copolymers and prodrug with a photocleavable junction points between the hydrophilic glycose and the hydrophobic P(αN_3_CL-*g*-alkyne)_*n*_ blocks were characterized through ^1^H NMR, FTIR, and GPC. The obtained copolymers and prodrug formed micelles with a spherical morphology in an aqueous solution. UV irradiation burst release of the encapsulated or conjugated drug. Cell viability was evaluated in response to these particles at concentrations of ≤1000 μg mL^−1^, with toxicity to HeLa cells when the concentration was ≧100 μg mL^−1^. After UV irradiation Gluco-ONB-P(αN_3_CL-*g*-PONBIMC)_10_ encapsulated DOX micelles entered and accumulated in the cells faster than its free-form counterpart and without UV irradiation. Thus, the results suggested that light-sensitive Gluco-ONB-P(αN_3_CL-*g*-PONBIMC)_10_ prodrug has potential targeted drug delivery applications.

## Conflicts of interest

There are no conflicts to declare.

## Supplementary Material
